# Micromass cultures are effective for differentiation of human amniotic fluid stem cells into chondrocytes

**DOI:** 10.6061/clinics/2018/e268

**Published:** 2018-03-24

**Authors:** Carolina Coli Zuliani, Mariana Freschi Bombini, Kleber Cursino de Andrade, Ronei Mamoni, Ana Helena Pereira, Ibsen Bellini Coimbra

**Affiliations:** I Reumatologia, Clinica Medica, Universidade Estadual de Campinas, Campinas, SP, BR; II Tocoginecologia, Universidade Estadual de Campinas, Campinas, SP, BR; III Imunologia, Patologia Clinica, Universidade Estadual de Campinas, Campinas, SP, BR; IV Laboratorio Nacional de Luz Sincrotron, Centro Nacional de Pesquisa em Energia e Materiais (CNPEM), Campinas, SP, BR

**Keywords:** Cartilage Repair, Chondrogenesis, Amniotic Fluid Mesenchymal Stromal Stem Cells, Micromass Culture

## Abstract

**OBJECTIVES:**

Articular cartilage is vulnerable to injuries and undergoes an irreversible degenerative process. The use of amniotic fluid mesenchymal stromal stem cells for the reconstruction of articular cartilage is a promising therapeutic alternative. The aim of this study was to investigate the chondrogenic potential of amniotic fluid mesenchymal stromal stem cells from human amniotic fluid from second trimester pregnant women in a micromass system (high-density cell culture) with TGF-β3 for 21 days.

**METHODS:**

Micromass was performed using amniotic fluid mesenchymal stromal stem cells previously cultured in a monolayer. Chondrocytes from adult human normal cartilage were used as controls. After 21 days, chondrogenic potential was determined by measuring the expression of genes, such as SOX-9, type II collagen and aggrecan, in newly differentiated cells by real-time PCR (qRT-PCR). The production of type II collagen protein was observed by western blotting. Immunohistochemistry analysis was also performed to detect collagen type II and aggrecan. This study was approved by the local ethics committee.

**RESULTS:**

SOX-9, aggrecan and type II collagen were expressed in newly differentiated chondrocytes. The expression of SOX-9 was significantly higher in newly differentiated chondrocytes than in adult cartilage. Collagen type II protein was also detected.

**CONCLUSION:**

We demonstrate that stem cells from human amniotic fluid are a suitable source for chondrogenesis when cultured in a micromass system. amniotic fluid mesenchymal stromal stem cells are an extremely viable source for clinical applications, and our results suggest the possibility of using human amniotic fluid as a source of mesenchymal stem cells.

## INTRODUCTION

Chondrocytes represent the only cell type present in articular cartilage and are responsible for its homeostasis 1. The cartilage extracellular matrix (ECM) is composed of a network, including collagens, proteoglycans and other smaller components. Collagen represents approximately 70-80% of the dry tissue weight of cartilage and ensures its strength and structural organization. Aggrecan is the second most important component of the ECM, and it provides the mechanical properties that allow cartilage to be compressed 2. Cartilage is known for its limited ability to repair or regenerate itself, which is due avascularity and a small number of cells with low mitotic activity and metabolism. Damage to cartilage may progress to osteoarthritis (OA), which can cause clinically affected individuals to experience pain and impair their joint function 3.

There have been numerous attempts to develop methods to assist in cartilage repair 4. One of these methods is cell therapy using high-density mobile systems (e.g., pellet or micromass culture) to induce chondrogenesis, the initial stage of cartilage formation 5. A previous study 6 reported that mesenchymal stem cells (MSCs) have the capacity to induce chondrocyte differentiation in pellet culture with serum-free medium containing glucocorticoids and transforming growth factor β (TGF-β).

For chondrogenesis, micromass culture provides a three-dimensional environment that allows cell–cell interactions similar to those during embryonic development; micromass was first used to study endochondral skeletal development in chicken embryos 7. Researchers 5 have compared the chondrogenic potential of MSCs from bone marrow (BM) in micromass or a pellet system and concluded that the micromass system is more suitable for inducing chondrogenesis. Our group studied chondrogenesis in MSCs from two different sources (periosteum-derived MSCs 8 and umbilical cord blood (UCB) cells) and concluded that micromass combined with TGF-β3 induces chondrogenesis in these two different populations 9.

Additionally, during chondrogenesis, MSCs acquire a spherical morphology and begin to express transcription factors, such as Sox9 10, Sox5 and Sox6, which regulate the genes encoding type II collagen, aggrecan, and other components of the ECM 11-13.

Other studies of horse MSCs from three different sources (UCB, amniotic fluid (AF) and BM) found that mitotic potential is greatest in MSCs collected from AF. Furthermore, these AF cells can be obtained from amniocentesis waste, and the absence of HLA-DR cell-surface receptors makes them immunologically advantageous for future clinical applications 14.

SCs (from UCB and AF) have also been analyzed using immunocytochemistry, and they express embryonic stem cell antigens, such as Oct-4, SSEA-4 and TRA-1-60, indicating pluripotency; moreover, SCs from AF likely represent an intermediate stage between embryonic and adult SCs 15. AF cells also express Tra-1-8 and the following germ layer markers: FGF-5 (an ectodermal marker), AFP (an endodermal marker) and Bra (a mesodermal marker). Injection of AF cells into immunodeficient mice does not result in tumor formation 16.

The aim of this work was to demonstrate that chondrogenesis can be induced in amniotic fluid mesenchymal stromal stem cells (AFMSCs) derived from pregnant women during their second trimester using a micromass system in the presence of TGF-β3 at both the gene expression and protein levels.

## MATERIALS AND METHODS

### 1. Collection of human amniotic fluid cells

After signing the informed consent form, 53 consecutive women undergoing amniocentesis during their second trimester of pregnancy allowed the collection of 30 ml of their AF. Amniocentesis was suggested by amniocentesis obstetrics upon suspicion of chromosomal abnormalities according to the Fetal Medicine-specific protocol, Hospital of Clinics, State University of Campinas (UNICAMP) (fetal structural anomalies detected on ultrasound and increased risk of chromosomal abnormalities by assessment of fetal risk). Amniocentesis was performed using ultrasound. The procedure was carried out as follows: the puncture area was cleaned and isolated using a fenestrated field; 2% lidocaine was applied to both the surface of the skin and subcutaneously without a vasoconstrictor; and the amniotic cavity was accessed by abdominal puncture using a 20G spinal needle for the withdrawal of 30 ml of AF in two 20-ml syringes. Ten ml of AF was subsequently sent to LABIMOCA (Laboratory of Molecular Biology of Cartilage, Faculty of Medical Sciences, UNICAMP) and cultured in 25 cm2 culture flasks containing 5 ml of α-Minimum Essential Medium (α-MEM, Gibco™, USA) with 20% fetal bovine serum (FBS, Gibco™ BR) and 1% Pen/Strep (Gibco™ USA). After 5 days, some AF cells had adhered to the flask. The supernatant and non-adherent cells were discarded, and the medium was replaced. At 70% confluence, the cells were harvested by treatment with 0.25% trypsin/EDTA (Gibco™, Can) for 5 min at 37°C and neutralized with 1% FBS.

The cells were washed twice with phosphate buffered saline (PBS) and centrifuged at 300 g for 10 min.

SCs were obtained from residual cells. After a normal karyotype result (performed in fetal medicine), approximately 5x104 cells were expanded in a 75 cm2 flask up to 70% confluence. The cells were then harvested, seeded in a 175 cm2 flask, and expanded up to 70% confluence. Next, we split the cells into three 175 cm2 flasks and expanded them up to 70% confluence. Samples with karyotype abnormalities were discarded. From the initial 53 samples, 12 presented normal karyotypes. Therefore, experiments were performed with the 12 normal samples.

### 2. Characterization of amniotic fluid mesenchymal stromal stem cells

In the fourth passage, some cells (approximately 1 to 3x105 per tube) were analyzed by flow cytometry for the expression of the following immunophenotypical markers: CD73, CD90, CD105, CD14, CD19, CD34, CD-45, HLA-DR, CD44, CD49c, CD151, SSEA-4, OCT-4, NANOG and CD117 (Biolegend™, USA and BD Pharmingen™). The cells were analyzed on a BD FACSCanto™ cytofluorometer using BD FACSDiva™ software (Becton, Dickson and Company, USA). During the same passage, pluripotency was assessed by plastic adherence (Olympus inverted optical microscope), as well as adipogenic, osteogenic and chondrogenic differentiation 17 by Stem Pro™ (Gibco, USA), according to the manufacturer’s protocol.

### 3. Differentiation

Cells were divided into 50 samples (each containing approximately 5x105 cells in a 20-µl volume) and placed into 96-well V-bottom culture plates. The cells were incubated at 37°C with 5% CO2 for 2 h to create micromass cultures. Next, medium was added without cell spreading. Differentiation was carried out using Dulbecco's Modified Eagle’s Medium (DMEM) supplemented with high glucose (Life Sciences), 10 ng/ml TGF-β3 (R&D Systems), 100 nM dexamethasone, 1x Insulin-transferrin-sodium selenite, linoleic-BSA (ITS+1) premix, 40 µg/ml proline, and 50 µg/ml ascorbic acid (Sigma-Aldrich, Poole, UK, http://www.sigmaaldrich.com). The cells remained in micromass culture for 21 days.

### 4. Confirmation of chondrocyte differentiation

#### 4.1. Histologic analysis

Histologic analysis of chondrogenesis was performed by hematoxylin-eosin (HE); Masson’s trichrome (MT) and picrosirius red (PR) were used for collagen-specific staining; and Alcian blue (AB) was used for proteoglycans. Immunochemistry analysis was performed using a rabbit polyclonal antibody (1:100, BIOSS™-USA, bs-0709R) for collagen type II and a rabbit polyclonal antibody (1:100, BIOSS™, USA, bs-11655R) for aggrecan; immunochemistry was performed as previously described by others 18.

#### 4.2. qRT-PCR: real-time quantitative polymerase chain reaction after reverse transcription

Chondrocyte differentiation was also confirmed by qRT-PCR. RNA was extracted using TRIzol™ remove contaminating genomic DNA. RNA was subsequently reverse transcribed to obtain cDNA using SuperScript II™ reverse transcriptase (Invitrogen). Quantitative PCR (real-time) was performed using the SYBR green method with a Stratagene Mx 3000P machine. For the genes type II collagen, aggrecan, SOX-9 and GAPDH, primers were designed from genomic libraries using the ABI Primer Express™ program (Applied Biosystems, USA). Each experiment was performed in triplicate. The primer sequences are listed in Table 1.

The total volume (12 µl) of each PCR reaction contained 6 µl SYBR Green PCR Master Mix, 10 ng cDNA (3 µl) and 150 pM (3 µl) of each forward and reverse primer. Real-time PCR reactions were performed under the following conditions: 95°C for 10 min (activation), 45 cycles of 95°C for 15 s, 60°C for 20 s, 72°C for 20 s (amplification), and 72°C for 1 min (final extension). Melting curves were examined after PCR was completed to confirm the specificity of the amplified products. Real-time PCR reactions were carried out with templates from MSCs without treatment (TO), MSCs under micromass conditions for 21 days with TGF-β3 (A1) and chondrocytes obtained from human adult articular cartilage (chondro).

### 5. Western blotting

Proteins secreted by cells into the medium during 21 days of micromass culture were precipitated by 2 mg/ml pepsin (Sigma-Aldrich, USA). The samples were incubated at 30°C for 30 min, stored overnight at 4°C with stirring, and centrifuged for 90 min at 2300 g. The pellets were washed twice with PBS. Protein was quantified using Bradford’s method. Thirty mg of protein was separated on a 10% sodium dodecyl sulfate polyacrylamide (SDS-PAGE) gel and handled according to the protocol followed by our laboratory 8,9 using a rabbit collagen II polyclonal antibody (1:500, BIOSS™, USA). Signals were visualized using a Super Signal West Pico Chemiluminescent Substrate Kit (Super Signal West Pico Stable Peroxide Solution, 500 ml; and Super Signal West Pico Luminol Enhancer Solution, 500 ml; Thermo Scientific Pierce Protein Products Research™).

### 6. Statistical analysis

Statistical analyses were carried out using one-way analysis of variance (ANOVA) with GraphPad Prism software. The confidence interval was 95% considering the multiple comparisons between groups. The results were considered significant at *p*<0.05.

## RESULTS

### 1. Amniotic fluid stem cell characterization

**Figure 1:**
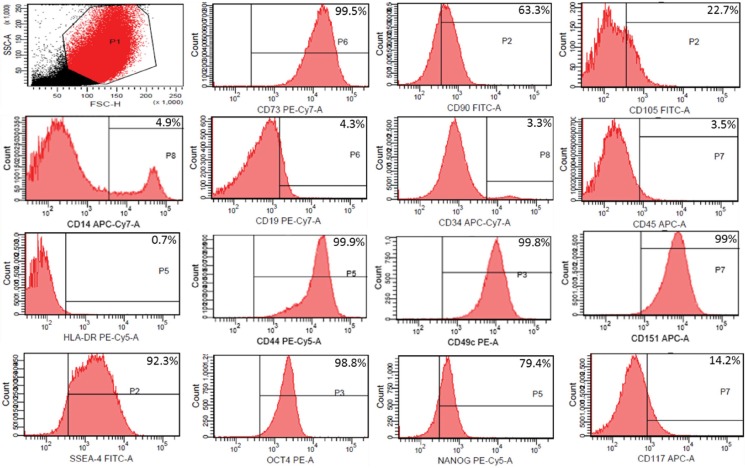
Flow cytometry analysis of the AFMSC pluripotency profile in samples at the third passage after harvesting. The percentage of each marker is shown in the upper right corner in the graphs.

**Figure 2:**
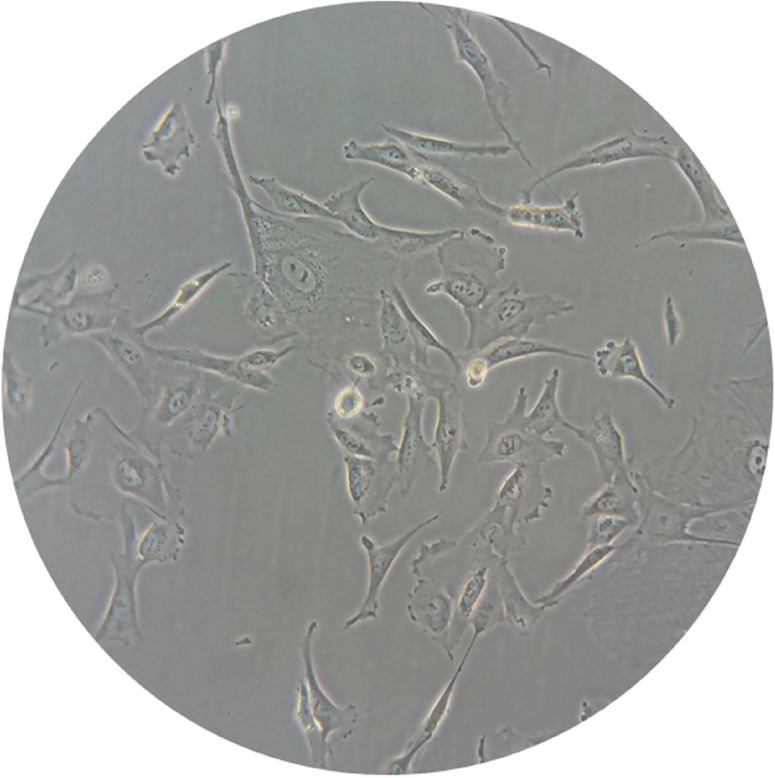
Adherent AFMSCs on plastic showing fibroblast-like morphology after 10 days in culture (400X).

**Figure 3:**
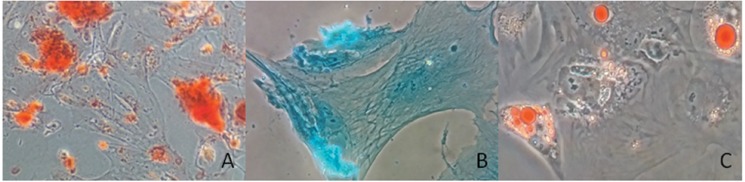
Capacity to differentiate: A) Osteogenic differentiation on plastic shown after staining with Alizarin red; B) Chondrogenic differentiation after staining with Alcian blue; C) Adipogenic differentiation shown by oil red O.

Immunophenotypic characterization by flow cytometry revealed that MSCs from human amniotic fluid (HAF) were positive for mesenchymal markers CD90 (63.3%), CD73 (99.5%), and CD105 (22.7%). MSCs were negative for the hematopoietic markers CD14 (4.9%), CD45 (3.5%), CD19 (4.3%), HLA-DR (0.7%) and CD 34 (3.3%). The following pluripotency markers investigated in this study were positive: SSEA-4 (92.3%), NANOG (79.4%), OCT4 (98.8%) and CD117 (14.2%). The chondrogenic potency markers CD49c (99.8%), CD44 (99.9%) and CD151 (99%) were also positive (Figure 1). Moreover, using Stem Pro (Gibco, NY), we observed plastic fixation (Figure 2) of AFMSCs, as well as osteogenic, adipogenic and chondrogenic differentiation (Figure 3).

### 2. Histology

**Figure 4:**
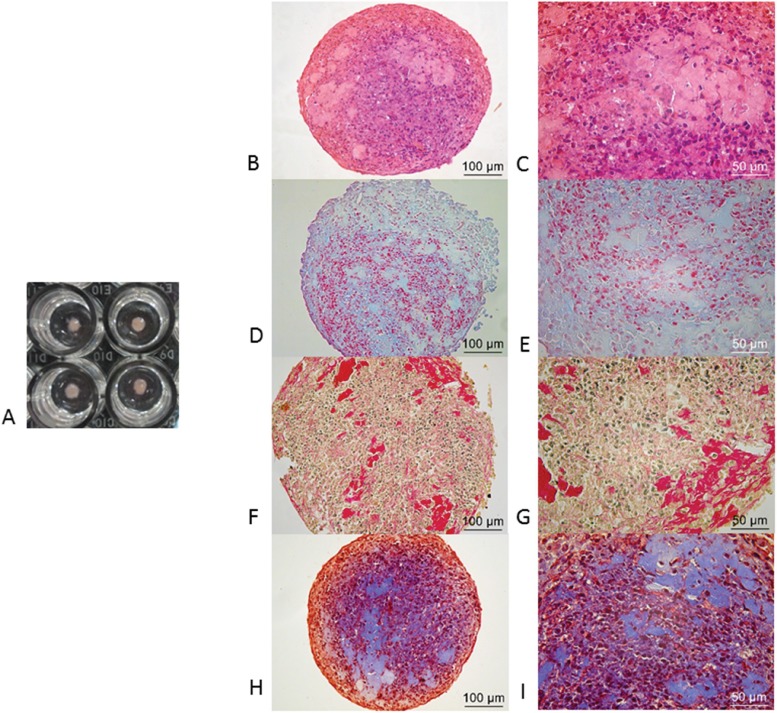
Histological analysis: A) Macroscopic aspect of micromasses after 21 days in TGF-β3-stimulated culture; B and C) The production of cartilage matrix is shown in pinkish and bluish staining (high amount of proteoglycans), and cell nuclei are shown in blue by HE; D and E) GAGs are shown in light blue by AB, and collagen production is shown in blue staining that permeated all the sections in red by MT; the cells contained black nuclei; F and G) In PR-stained sections, collagen appears pale yellow but becomes red when collagen fibers aggregate; H and I) In MT-stained sections, collagen production appears in blue staining that permeated all the sections in red; the cells contained black nuclei.

Micromasses were fixed in 4% buffered formalin and rinsed twice in PBS. Next, the micromasses were processed and embedded in paraffin. After preparation, 5-µm-thick sections were stained with HE, MT, PR and AB. The results are shown in Figure 4. HE shows the production of cartilage matrix in pinkish and bluish staining (high amount of proteoglycans), and cell nuclei appear in blue. Collagen production is shown by MT in blue staining that permeated all the sections in red; the cells contained black nuclei. In PR-stained sections, collagen appears pale yellow to light brown; when several collagen fibers aggregate, they appear red. Glycosaminoglycans (GAGs) appear light blue with AB staining.

### 3. Real-time PCR (qRT-PCR)

**Figure 5:**
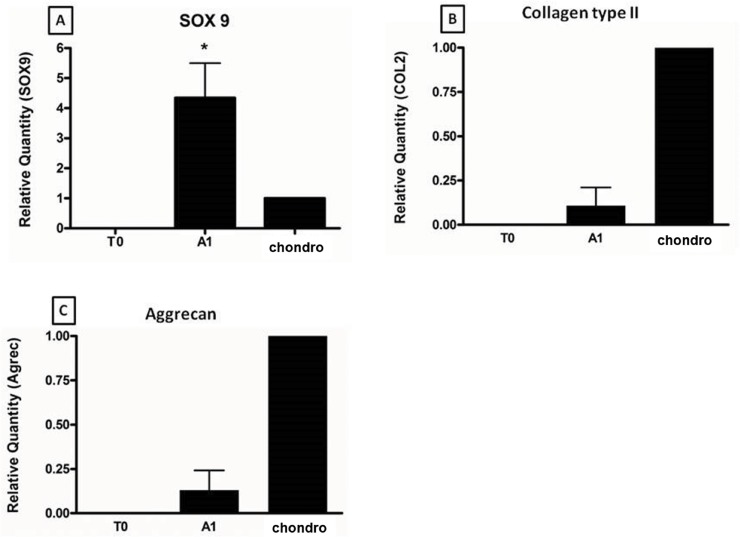
qRT-PCR: T0 represents the negative control for AFMSC gene expression; A1: Newly differentiated cell gene expression; chondro represents adult human articular chondrocytes as a positive control. A) SOX-9 expression is higher in young cells than in adult cells. B) and C) Type II collagen and aggrecan are expressed in new cells but not in AFMSCs, confirming that differentiation occurred.

We confirmed the expression of cartilage-specific genes in differentiated cells. Figure 5A shows that the expression of SOX9 in cells subjected to micromass culture for 21 days with TGF-β3 (A1) was significantly higher than that in cells in monolayer culture without TGF-β3 (TO) and normal human articular cartilage (chondro) (*p*<0.05). The expression of type II collagen in cells subjected to micromass culture for 21 days with TGF-β3 (A1) (Figure 5B) was higher than that in cells in monolayer culture without TGF-β3 (TO), which were used as a negative control, and lower than that in normal human articular cartilage (chondro), which was used as a positive control. Figure 5C shows that the expression of aggrecan in cells during micromass culture for 21 days with TGF-β3 (A1) was higher than that in cells in monolayer culture without TGF-β3 (TO; negative control) and lower than that in normal human articular cartilage (chondro; the positive control).

### 4. Western blotting

**Figure 6:**
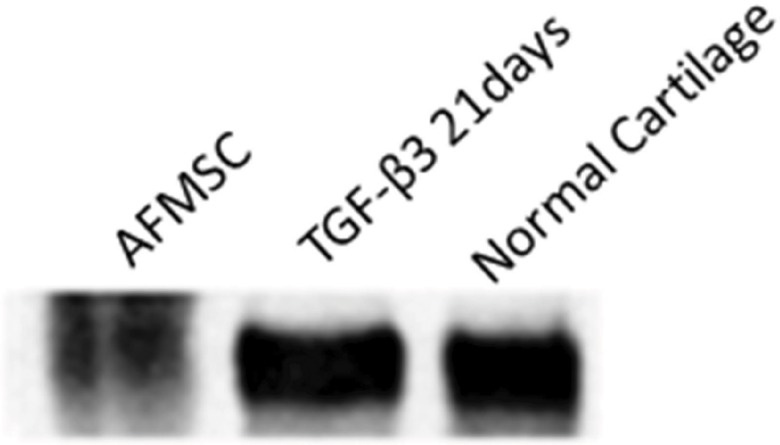
Western blotting showing positivity for collagen type II in culture medium with TGF-β3 after 21 days.

As shown in Figure 4, collagen type II, which is very specific to articular cartilage chondrocytes, was produced and secreted by cells into the medium during micromass culture in the presence of TGF-β3 for 21 days. Collagen type II was detected in pooled medium (Figure 6).

### 5. Immunohistochemistry

**Figure 7:**
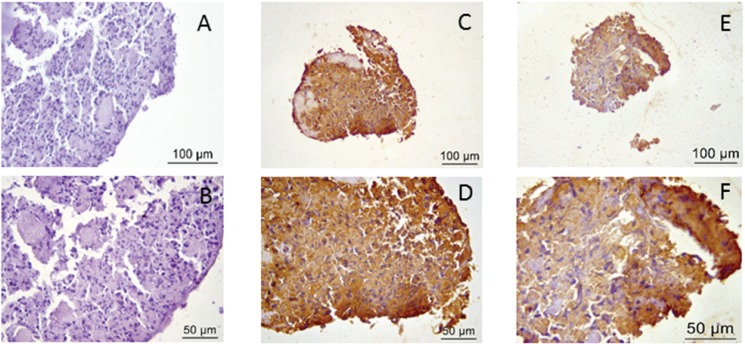
Immunohistochemistry. A and B) Negative control; C and D) Positive immunohistochemical staining for collagen type II; E and F) Positive immunohistochemical staining for aggrecan.

In Figure 7, immunohistochemical staining for both collagen type II and aggrecan is shown and compared with negative staining.

## DISCUSSION

Articular cartilage has a limited capacity to recover from trauma. There have been numerous attempts to protect and/or rehabilitate injured cartilage tissue, but these medical procedures do not guarantee its regeneration. The methods used to correct problems with cartilage include microfracture, chondroplasty, osteochondral grafts 19,20, abrasion arthroplasty 13 and autologous chondrocyte implantation (ACI), a surgical procedure widely used to regenerate articular cartilage 21. However, despite the new materials currently available, ACI has certain disadvantages, including the possibility of chondrocyte leakage and death, as well as the potential for fibrocartilage production in some patients 22. Compared with other therapies, therapy using MSCs derived from human AF is a promising new treatment because it has great potential for tissue recovery, given the chondrogenic potential and pluripotency of these SCs 16.

In the present study, the immunophenotypic characterization of MSCs from HAF was slightly different from that of MSCs from other sources. This difference is likely due to the high variability of cell lineages present in AF, which has been observed by others 23,24.

We studied the effects of micromass culture on MSCs derived from human AF over a period of 21 days. We chose to study the micromass system because previous studies 5 have shown that compared with the pellet system, this system yields a higher sulfate GAG (sGAG) deposition rate and higher overall dry weight. In micromass systems, new cells appear to be more homogeneous, and apoptosis is lower in these cells than in cells differentiated using the pellet system. Finally, the expression of type II collagen is significantly higher than that of type X and type I collagen, which are indicators of fibrocartilage. Thus, the micromass culture system appears to be the most suitable for inducing chondrogenesis 5. The results we observed are similar to those previously described, despite the fact that we observed the protein expression of type II collagen on day 21 (using western blotting), while previous studies reported collagen production on day 14 (using immunohistochemical techniques). This discrepancy might be related to differences in study design. Additionally, qRT-PCR revealed elevated expression of type II collagen, which was consistent with chondrogenesis. Conversely, type II collagen gene expression was not detected using cDNA from AFMSCs without differentiation. For the first time, we also compared the expression of type II collagen in “newborn” cells with that in normal human adult cartilage, and we confirmed similar expression levels between the two cell populations. By contrast, type II collagen expression was not observed in AFMSCs that had not undergone differentiation. This finding reinforces the fact that the micromass system provides an efficient method for inducing chondrogenesis. Our evidence also complements the findings of another study 5 in which the authors examined the expression of SOX-9 and aggrecan up to day 14. We continued culturing cells until day 21 and analyzed the gene expression of SOX-9 and aggrecan; moreover, we measured the levels of these genes in normal human adult cartilage, which yielded results that are more relevant to human physiology.

When chondrogenesis is induced in AFMSCs using the pellet system technique, the expression of type II collagen is higher in cells treated with TGF- β3 than in cells treated with TGF-β1 or TGF-β2 25. Interestingly, cells treated with TGF-β1 produce significantly higher amounts of sGAG than do cells supplemented with TGF-β3 26. However, the expression of sGAG is higher in AFMSCs stimulated with TGF-β3 or TGF-β2 and dexamethasone than in those stimulated with dexamethasone and TGF-β1. Thus, TGF-β3 appears to be superior to TGF-β1 25. Our decision to use TGF-β3 was also based on a previous study from our group in which TGF-β3 was more efficient for inducing differentiation of MSCs derived from UCB into chondrocytes 9 than other growth factors (TGF-β3 was combined with dexamethasone to induce chondrogenesis). As a result, we were able to successfully induce the expression of type II collagen, aggrecan and SOX-9.

In this study, chondrogenesis was induced in HAF-derived MSCs stimulated with TGF-β3 over 21 days using the micromass system. We confirmed this differentiation by analyzing the expression of the most important genes for the formation of articular cartilage (SOX-9, collagen type II and aggrecan) and confirmed the production of collagen type II at the protein level using histology or western blotting. Compared with normal human cartilage (used as a standard), cells under the micromass system showed significantly higher expression of the SOX-9 gene, as depicted in Figure 3A. This result indicates that it is possible to reproduce human chondrogenesis in vitro, as SOX-9 is a key factor in inducing this process. Our results are reinforced by another study 5 in which the authors detected SOX-9 expression in cells under the micromass system but did not find a significant difference compared with that in cells under the pellet system, thereby indicating that transcription factor SOX-9 is essential for inducing chondrogenesis in both high-density systems.

Furthermore, Arnhold S et al. 27,28 demonstrated that HAF contains a heterogeneous cell population composed of many cell types, including fibroblast cells with the capacity to differentiate into osteogenic, adipogenic, and chondrogenic lineages; epithelial cells; and cell types that could become other cell lineages. Thus, this source of SCs can potentially be applied to diseases of osteogenic and chondrogenic lineages, as well as neurodegenerative diseases, such as retinopathy of prematurity.

Our results support advancement from preclinical to clinical research targeting the implantation of new chondrocytes differentiated from AFMSCs under the micromass culture system in cartilage lesions. Moreover, our findings support the use of newly differentiated chondrocytes on scaffolds in future studies.

This novel combination of MSCs derived from HAF using the micromass culture system and TGF-β3 over a period of 21 days demonstrates an increase in chondrogenesis and the induction of the expression of SOX-9, collagen type II and aggrecan.

## AUTHOR CONTRIBUTIONS

Zuliani CC and Bombini MF contributed equally to this work, they were responsible for the data collection and/or assembly, data analysis and interpretation, and manuscript writing. Andrade KC was responsible for the provision of study material or patients. Pereira AH and Mamoni R were responsible for the data analysis and interpretation. Coimbra IB was responsible for the study conception and design, financial support, manuscript writing, and approval of the final version of the manuscript.

## Figures and Tables

**Table 1:** Sequences of primers used in qRT-PCR reactions were designed using the ABI Primer Express program (Applied Biosystems, USA).

Collagen II Forward	GGCAATAGCAGGTTCACGTACA
Collagen II Reverse	CGATAACAGTCTTGCCCCACTT
Aggrecan Forward	TCGAGGACAGCGAGGCC
Aggrecan Reverse	TCGAGGGTGTAGCGTGTAGAGA
GAPDH Forward	GCACCGTCAAGGCTGAGAAC
GAPDH Reverse	CCACTTGATTTTGGAGGGATCT
